# Safety of tildrakizumab: a disproportionality analysis based on the FDA adverse event reporting system (FAERS) database from 2018–2023

**DOI:** 10.3389/fphar.2024.1420478

**Published:** 2024-07-10

**Authors:** Jinger Lin, Xiangqi Chen, Min Luo, Qianwei Zhuo, Haosong Zhang, Nuo Chen, Yunqian Zhuo, Yue Han

**Affiliations:** ^1^ Department of Dermatology, The Union Hospital, Fujian Medical University, Fuzhou, Fujian, China; ^2^ Department of Dermatology, 900Th Hospital of Joint Logistics Support Force, Chinese People's Liberation Army, Fuzhou, Fujian, China; ^3^ Department of Dermatology, Fuzhou First General Hospital, Fuzhou, Fujian, China

**Keywords:** plaque psoriasis, tildrakizumab, adverse drug reactions, disproportionality analysis, FAERS

## Abstract

**Background:** Tildrakizumab, the IL-23 inhibitor, is used to treat plaque psoriasis and psoriatic arthritis. Many studies have reported adverse drug reactions (ADRs) associated with Tildrakizumab.

**Objective:** The aim of this study was to describe ADRs associated with Tildrakizumab monotherapy by mining data from the U.S. Food and Drug Administration Adverse Event Reporting System (FAERS).

**Methods:** The signals of Tildrakizumab-associated ADRs were quantified using disproportionality analyses such as the reporting odds ratio (ROR), the proportional reporting ratio (PRR), the Bayesian confidence propagation neural network (BCPNN), and the multiitem gamma Poisson shrinker (MGPS) algorithms.

**Results:** A total of 10,530,937 reports of ADRs were collected from the FAERS database, of which 1,177 reports were identified with tildrakizumab as the “primary suspect (PS)”. Tildrakizumab-induced ADRs occurred against 27 system organ classes (SOCs). A total of 32 significant disproportionality Preferred Terms (PTs) conformed to the algorithms. Unexpected significant ADRs such as coronavirus infection, herpes simplex, diverticulitis, atrial fibrillation and aortic valve incompetence were also possible. The median time to onset of Tildrakizumab-associated ADRs was 194 days (interquartile range [IQR] 84–329 days), with the majority occurring, within the first 1 and 3 months after initiation of Tildrakizumab.

**Conclusion:** This study identified a potential signal for new ADRs with Tildrakizumab, which might provide important support for clinical monitoring and risk prediction.

## 1 Introduction

Psoriasis is a chronic inflammatory papulosquamous skin disease that affects approximately 60 million adults and children and is carries an increased risk of comorbidities, such as depression, psoriatic arthritis, cardiovascular disease, type 2 diabetes, obesity, and hypertension (Kaushik and Lebwohl, 2019; Ghoreschi, 2021; Sbidian et al., 2022). Common clinical types of psoriasis include psoriasis vulgaris and chronic plaque psoriasis. Other relatively rare types include guttate, erythrodermic, and pustular psoriasis. In Europe and North America, the prevalence of chronic plaque psoriasis is approximately 2%. Additionally, about 40% of individuals with pemphigus vulgaris develop chronic plaque psoriasis over time (Reich et al., 2017; Griffiths et al., 2021). Roughly 20% of patients with plaque psoriasis present with moderate to severe forms, affecting more than 10% of the body surface area or involving areas such as the scalp, face, joints, and nails (Narcisi et al., 2023). The pathogenesis of psoriasis is not fully understood, although immune dysregulation and genetic factors play a crucial role. Previous studies have highlighted the important role of the interleukin (IL)-23/IL-17 axis in the pathogenesis of psoriasis. This axis involves IL-23 secreted by macrophages and dendritic cells and IL-17 secreted by T-cells. It promotes epidermal proliferation, thickening of the stratum corneum, and affects angiogenesis leading to immune cell infiltration (Sabat et al., 2019).

IL-23 consists of the p40 and p19 subunits linked by a disulfide bond. It shares the p40 subunit with IL-12, and this subunit is also targeted in ustekinumab treatment. IL-23 promotes the expansion and differentiation of Th17 cells and the secretion of IL-17, which is mainly produced by activated T-cells. In addition, IL-23 upregulates CCL20, a chemokine that contributes to the movement of Th17 cells. IL-23 has a strong synergistic effect with TNF-α, IL-22, and IFN-γ, and is a key factor in the pathogenesis of arthritis and inflammatory bowel disease (Stritesky et al., 2008; Markham, 2018; Yang et al., 2021). Mild psoriasis is usually treated with steroids and vitamin D analogues, while moderate to severe psoriasis is treated with Phototherapy, systemic immunosuppressants, small molecules, and biologics (Mason et al., 2019; Ghoreschi et al., 2021). In some countries, concomitant use of biologics, oral medicine, or phototherapy may be considered for moderate-to-severe plaque psoriasis (Griffiths et al., 2021).

Tildrakizumab (TIL), is a high-affinity human IgG1/K monoclonal antibody, that selectively targets the p19 subunit of IL-23, thereby blocking both IL-23 activity and its receptor binding. In March 2018, the US Food and Drug Administration’s (FDA) approved to TIL for the treatment of moderate-to-severe chronic plaque psoriasis, either alone or in combination with systemic therapy or phototherapy (Galluzzo et al., 2022). The recommended dosage regimen for TIL administered via subcutaneous injection is 100 mg at week 0, week 4, and subsequently every 12 weeks thereafter (Pharma, 2022), and European countries also allow treatment with 200 mg in special circumstances, such as high disease burden or body weight ≥90 kg (Reich et al., 2020). A study involving 24 patients with moderate-to-severe plaque psoriasis showed that after treatment with TIL, 85.72% of patients achieved a PASI score of 75, and 80.9% had a PASI score of ≤3 at week 12, which suggests the efficacy of TIL for short-term treatment of chronic plaque psoriasis (Galán-Gutierrez et al., 2022). The large-scale randomized controlled phase III trials reSURFACE1 and reSURFACE2 (ClinicalTrials.gov identifiers: NCT01722331; NCT01729754) contributed significantly to the introduction of TIL, compared with placebo and etanercept^5^. The drug has shown significant efficacy in the treatment of chronic plaque psoriasis. This assertion is further supported by the study of Mastorino et al. (Mastorino et al., 2022). Additionally, TIL has shown efficacy in the treatment of psoriatic arthritis, nail psoriasis, and scalp psoriasis (Onuora, 2021; Bardazzi et al., 2023). However, common adverse drug reactions (ADRs) like nasopharyngitis, headache, upper respiratory tract infections, and psoriasis exacerbations should not be ignored during the course of medication. In addition, in the open-label, phase IV RIBUTE study, adult patients with moderate-to-severe psoriasis treated with TIL frequently developed neurological disorders such as headache, along with infections, infestations, and gastrointestinal disorders (Costanzo et al., 2023). Therefore, given that TIL has been on the market for 5-year, it is important to scrutinise its ADRs using data mining algorithms for pharmacovigilance. Furthermore, conducting short-term experimental studies and meta-analyses on the safety of the drug will also provide valuable insights.

The FDA Adverse Event Reporting System (FAERS) database, is the world’s largest repository of pharmacovigilance information and facilitates spontaneous reporting of ADRs and medication errors by various stakeholders including drug manufacturers, consumers, healthcare professionals, physicians, pharmacists, and others, stands as the world’s largest repository of pharmacovigilance information. It greatly facilitates the FDA’s comprehensive assessment of drug safety after a drug is marketed (Hosoya et al., 2021; Yin et al., 2022). Our findings have potential to provide physicians and health policymakers with meaningful insights in monitoring TIL-associated ADRs, thereby fostering rational use of clinical medications.

## 2 Method

### 2.1 Data sources and cleaning

We conducted a retrospective pharmacovigilance study using the FAERS database (https://fis.fda.gov/extensions/FPD-QDE-FAERS/FPD-QDE-FAERS.html) of patients using TIL from the first quarter (Q1) of 2018 to the fourth quarter (Q4) of 2023, since TIL was approved for use by the FDA in March 2018, FAERS covers seven areas: demographic and administrative information (DEMO), drug information (DRUG), adverse drug reaction information (REAC), patient outcome information (OUTC), reporting source information (RPSR), date of treatment initiation and date of end of reported medication (THER) and medication administration indications (INDI) (Yu et al., 2021; Zheng et al., 2023).

ADRs in the FAERS database are coded according to Preferred Terms (PTs) in the Medical Dictionary for Regulatory Activities Code 24.0 (MedDRA), and multiple PTs can correspond to a System Organ Class (SOC) level (Tian et al., 2022; Wu et al., 2023). We used four algorithms to classify SOCs.We analysed SOC and PTs by four algorithms to assess the associations between drug-related ADRs and drugs. Only reports documenting TIL as “primary suspect (PS)” drug were included in our analyses. First, the FAERS database was searched using use brand names and generic names, including “TILDRAKIZUMAB”,“IIUMYA”, “SCH900222”, “SCH-900222”, “MK-3222”, “BLINDED TILDRAKIZUMAB 100 MG ML INJECTION”, “BLINDED TILDRAKIZUMAB INJECTION”, “BLINDED TILDRAKIZUMAB”, “TILDRAKIZUMAB ASMN”, “ILUMYA TILDRAKIZUMAB”, “ILUMYA TILDRAKIZUMAB ASMN” and “ILUMYA TILDRAKIZUMAB ASMN” as PT names and sorted them in order of CASEID, FDA_DT, and PRIMARYID, in that order Duplicate PRIMARYIDs were eliminated and the one with the largest PRIMARYID value, was taken to extract information about the patient’s relevant characteristics, and the information was integrated using Microsoft Excel software. In addition, non-drug related issues such as “product administration interrupted”, “product administration error”, “product distribution issue”, “product storage error”, “product dose omission issue”, “circumstance or information capable of leading to medication error”, “treatment noncompliance”, “needle issue”, “inappropriate schedule of product administration” etc., were also excluded from the PTs, and “product used for unknown indication” was excluded from the indications section.

### 2.2 Disproportionality analysis and signal detection

Disproportionality analysis refers to the assessment of spontaneously reported cases to detect signals in order to hypothesise a potential causal relationship between a specific drug and its ADRs, using all ADRs as a statistical background (Zou et al., 2023; Li et al., 2024). The detected signals indicate an association between medications and ADRs, The detected signals indicating an association between medications and ADRs can be quantitatively identified by two main statistical methods and four algorithms: 1). Frequentist: reporting odds ratios (ROR) and proportional reporting ratio (PRR); 2). Bayesian: Bayesian confidence propagation neural network (BCPNN) and multi-item gamma Poisson shrinker (MGPS). ROR and PRR have the advantages of early detection, high signal sensitivity, and simple calculation of the signal value, whereas BCPNN and MGPS are more conservative in terms of signal detection, with a high level of specificity, which reduces the generation of false positive signals (Chen et al., 2008; Noguchi et al., 2021). Among these four algorithms, ROR algorithm produces the highest number of signals with the strongest evidence, with a higher ROR ratios indicating a more robust signals (Sakaeda et al., 2013; Sakaeda et al., 2014). Therefore, we believe that signal detection should be based on the ROR algorithm as the minimum standard, when the more algorithms are satisfied, the higher the robustness and significance. Thus, we define a signal as positive if it meets the ROR algorithm criteria and significant if it meets the criteria of two or more algorithms. The criteria for the four algorithms were as follows: ROR required a number of reported cases (N) ≥ 3 and a lower limit of the 95% confidence interval (CI) > 1. PRR required a PRR ≥2, χ^2^ ≥ 4 and N ≥ 3. BCPNN requires IC025 (lower limit of the 95% CI for the IC) > 0, and MGPS requires an EBGM05 (lower limit of the 95% CI for the EBGM) > 2. Calculations for each algorithms were based on a 2x2 columnar table (Supplementary Table S1), and detailed algorithm and criteria are shown in Supplementary Table S2 ((Tian et al., 2022), (Fang et al., 2023)).

**FIGURE 1:**
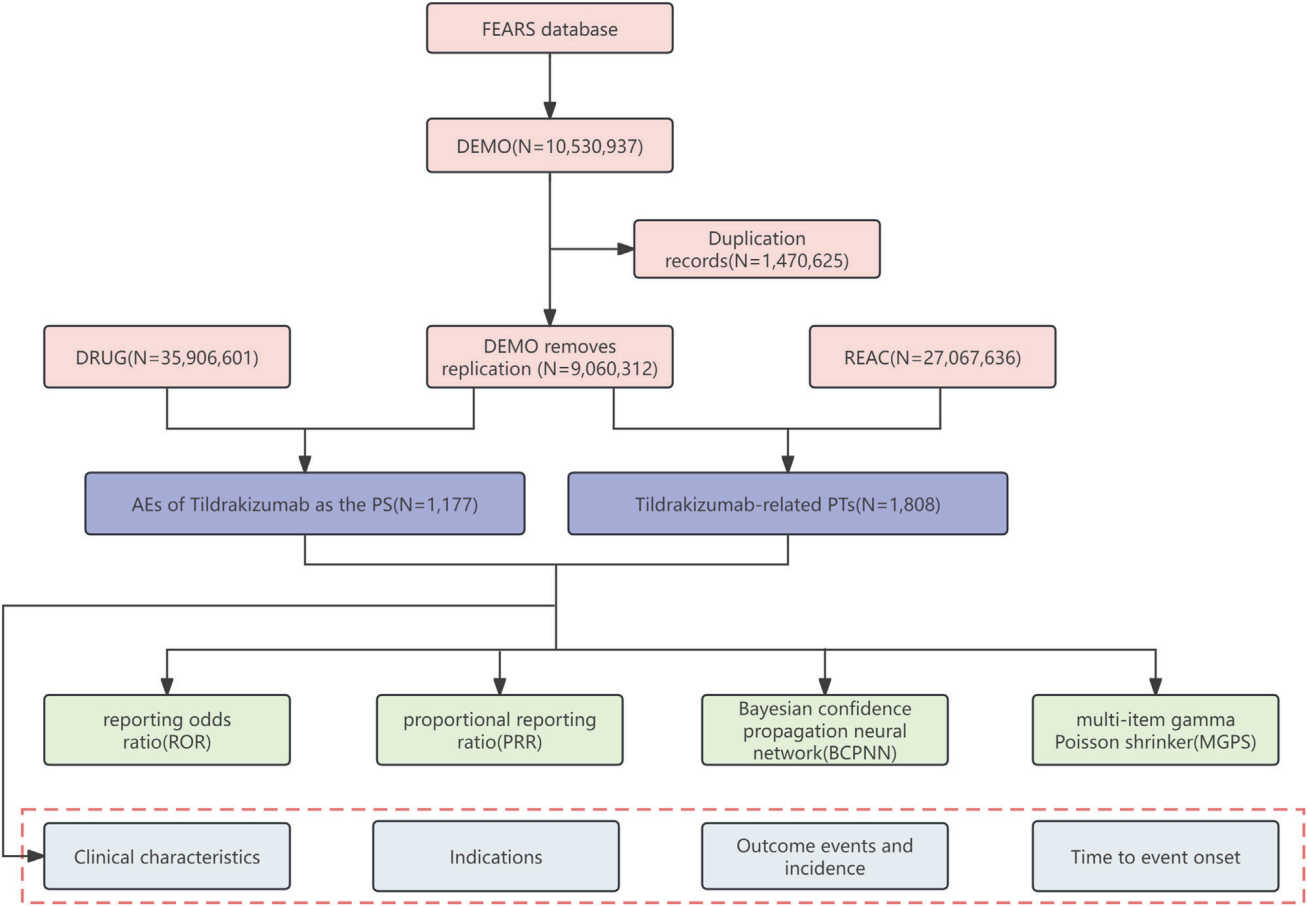
Flow diagram (DEMO demographic and administrative information, DRUG drug information, REAC preferred terminology for adverse event, PS primary suspect drug, PTs preferred terms).

Time-to-event onset (TTO) indicates the time interval between the date of administration and the occurrence of ADRs. Reports containing input errors (ADRs occurring before administration) or inaccurate date input were excluded. We used the Kruskal–Wallis test to determine if there were differences in the median time of positive SOCs and PTs to observe the variations in different ADRs. Additionally, we conducted the Kolmogorov-Smirnov Goodness of Fit test and Weibull test on TTO (Quinn and Quinn, 2010; Hazra and Gogtay, 2016). A *p*-value >0.05 indicated that the data complied with the Weibull test in the Kolmogorov-Smirnov Goodness of Fit tests. The Weibull tests include shape parameter (β) and scale parameter (η), which can further explain the trend of ADRs and describe the characteristic time of occurrence for approximately 63.2% of ADRs. The flowchart is shown in Figure 1. All analyses were performed using R software version 4.2.2.

## 3 Results

### 3.1 Baseline characteristics

**FIGURE 2:**
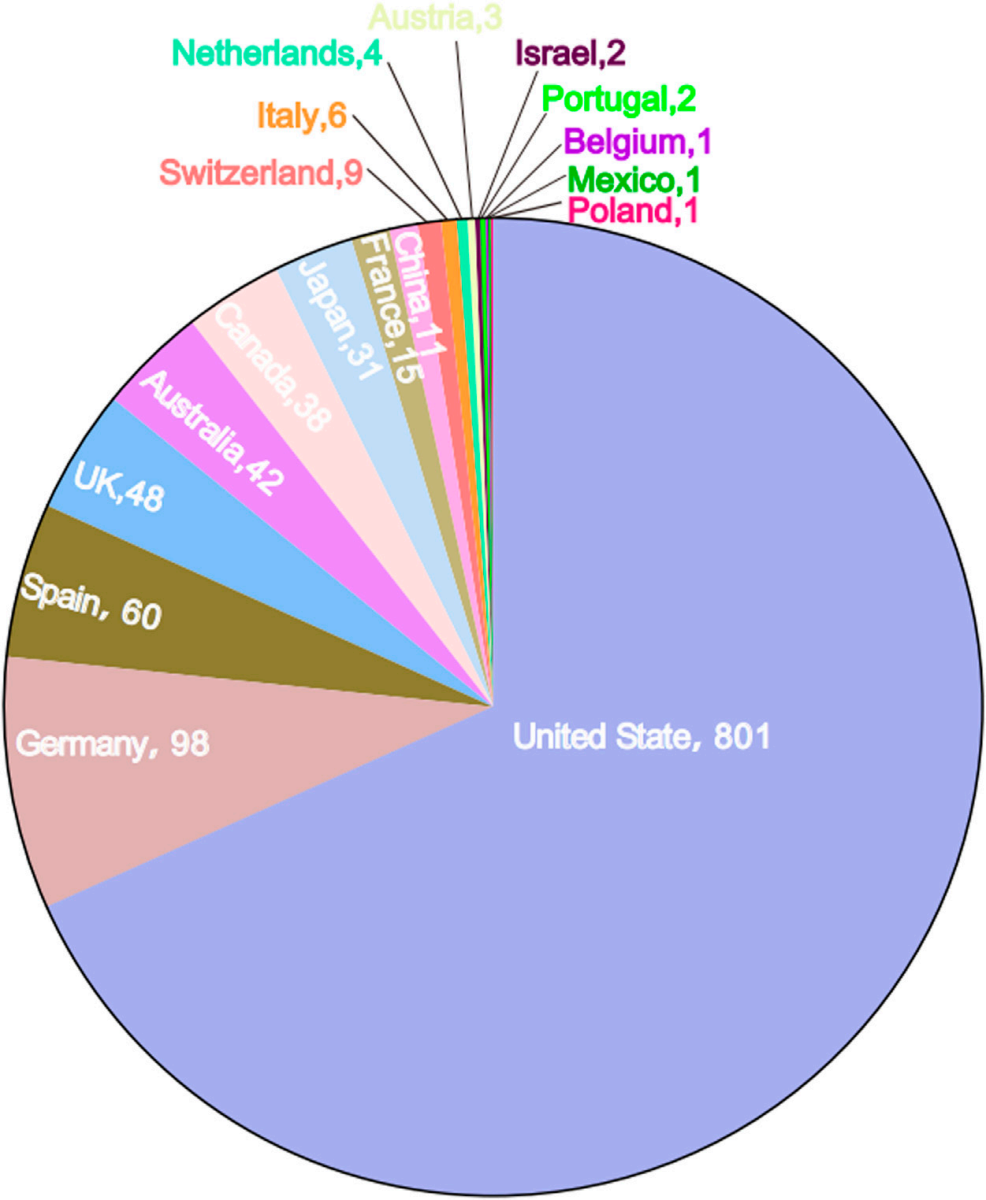
Number of Tildrakizumab-related ADR reports by country in the data table. The name of the country and the number of cases report are indicated.

From the first quarter (Q1) of 2018 to the fourth quarter (Q4) of 2023, a total of 1,177 reports of TIL as the primary suspect drug were identified in the FAERS database, excluding duplicate reports (Table 1). A higher proportion of reports were made by males (44.1%) than females (39.9%), and the missing data rate was 16.0%. Patients aged 18–64 years accounted for 25.1% of the total number of reports, and patients weighing between 50–100 kg accounted for 14.0%. The majority of ADR reports came from consumers (52.2%) and health professionals (25.4%). Geographically, the United States had the highest number of ADR reports (68.1%), followed by Germany (8.3%), Spain (5.1%), the United Kingdom (4.1%), and Australia (3.2%). Countries in Asia and Africa contributed to Asian and African countries accounted for a smaller proportion of the reporting population, as shown in Figure 2. Serious ADRs-related to TIL mainly included other significant medical events (28.5%) and hospitalization—initial or prolonged (17.1%), with 40 (3.4%) deaths, 18 (1.5%) life-threatening injuries and in 8 (0.7%) disabilities.

**TABLE 1:** Clinical characteristics of patients with TIL ADRs from the FAERS database.

Characteristics	TIL (N = 1,177)	
	Case reports(N)	Proportion (%)
Gender		
Female	470	39.9
Male	519	44.1
Unknown	188	16.0
Age (year)		
18–64	295	25.1
65–85	189	16.1
≥86	10	0.8
Unknown	683	58.0
Weight (kg)		
<50	7	0.6
>100	76	6.5
50–100	165	14.0
Unknown	929	78.9
Reporter		
Consumer	614	52.2
Health professional	299	25.4
Physician	193	16.4
Pharmacist	36	3.1
Other health-professional	9	0.8
Unknown	26	2.2
Reported countries (top five)		
The United States	801	68.1
Germany	98	8.3
Spain	60	5.1
United Kingdom	48	4.1
Australia	42	3.2
Serious outcomes		
Other serious important medical event	335	28.5
Hospitalization-initial or prolonged	201	17.1
Death	40	3.4
Life-threatening	18	1.5
Disability	8	0.7
Congenital anomaly	2	0.2
Unknown	687	58.3
Indications (top five)		
Psoriasis	642	54.5
Hypertension	71	6.0
Diabetes mellitus	20	1.7
Psoriatic arthropathy	19	1.6
Depression	9	0.8
Reporting year(y)		
2018	13	1.1
2019	96	8.2
2020	168	14.3
2021	219	18.6
2022	289	24.6
2023	392	33.3

TIL, tildrakizumab; ADRs, adverse drug reactions; Unknown indicates missing data, are not included in the subsequent calculation.

### 3.2 Disproportionality analysis

**FIGURE 3:**
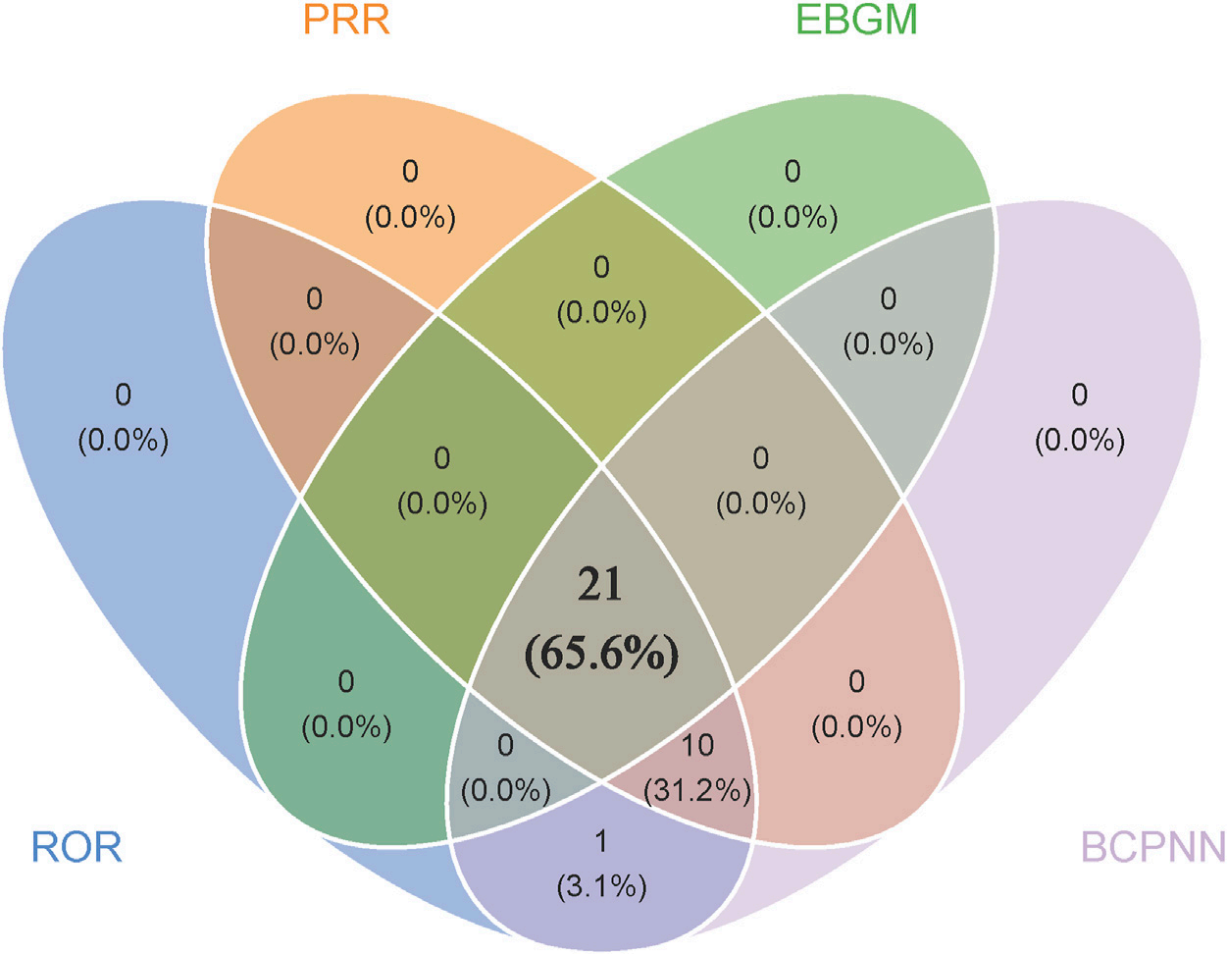
Venn that 21 PTs satisfy all four algorithms simultaneously. Blue module represents ROR, orange module represents PRR, green module represents EBGM and purple module represents BCPNN.

**FIGURE 4:**
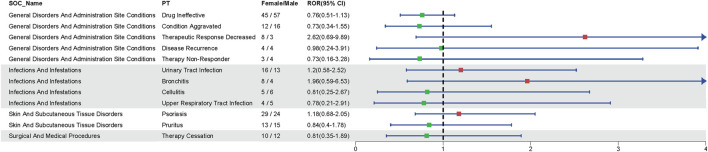
Gender-related ADRs. Reporting odds ratio (ROR) and 95% CI for all significant gender-related ADRs.Green and red dots represent the ROR.

Disproportionality analyses of ADRs with TIL were initially performed at the SOC level. We identified 27 organ systems affected by TIL-related ADRs (Table 2). “Surgical and medical procedures” satisfied all four algorithms and has the highest values for each. This SOC reports the least amount of medical expertise required. “Infections and infestations” met the ROR and PRR algorithms, indicating high sensitivity and early adverse reactions to TIL. The remaining four SOCs met only the ROR algorithm criteria, as shown in bold in Table 2. The lack of positive signals in PRR, BCPNN, and EBGM suggests that these signals are more ambiguous and less specific in the TIL user group. Table 3 shows 32 clearly disproportionate PTs, of 21 PTs met all four algorithms simultaneously (Figure 3). Previous studies on TIL have shown that upper respiratory tract infection (PT: 170715085), respiratory tract infection (PT: 228041992), injection site urticaria (PT: 163513461), psoriasis (PT: 172351161), pruritus (PT: 175510751), rash (PT: 157440671), and PTs such as myocardial infarction (PT: 222780722) were consistent with the results of clinical trials (Galluzzo et al., 2022). Some of these PTs showed high RORs, including vulvovaginal candidiasis (ROR = 121.70, PT:213490533), herpes simplex (ROR = 18.48, PT: 201335143), and aortic valve incompetence (ROR = 31.99, PT: 203349819). After further grouping by gender, we observed that diminished response to treatment, urinary tract infection, bronchitis and psoriasis were more common in females, whereas drug ineffectiveness, aggravated condition recurrence, therapy non-response, cellulitis, upper respiratory tract infection, pruritus, and therapy cessation were more prevalent in males (Figure 4; Supplementary Table S3). Although the *p* values were not significant, more cases in the male and female subgroup may be needed to show differences. Further analysis will be necessary as more reports are submitted.

**TABLE 2:** Signal strength of ADRs of TIL at the system SOC level in FAERS database.

SOC	N*	ROR (95% CI)	PRR (χ^2^)	IC (IC025)	EBGM (EB05)
Surgical and medical procedures^a^ ^,^ ^b^	98	3.43 (2.80–4.20)^c^	3.32 (160.93)^c^	1.73 (0.06)^c^	3.32 (2.71)^c^
Infections and infestations^a^	255	2.33 (2.04–2.65)^c^	2.17 (170.12)^c^	1.12(−0.55)	2.17 (1.90)
Injury, poisoning and procedural complications^a^	390	1.66 (1.48–1.85)^c^	1.54 (82.72)	0.62 (−1.05)	1.53 (1.38)
Skin and subcutaneous tissue disorders^a^	190	1.56 (1.34–1.81)^c^	1.51 (34.71)	0.59 (−1.07)	1.51 (1.30)
Cardiac disorders^a^	57	1.34 (1.03–1.74)^c^	1.33 (4.74)	0.41 (−1.26)	1.33 (1.02)
General disorders and administration site conditions^a^	410	1.12 (1.01–1.25)^c^	1.10 (4.32)	0.13 (−1.53)	1.10 (0.99)
Neoplasms benign, malignant and unspecified (incl cysts and polyps)	79	1.06 (0.85–1.33)	1.06 (0.28)	0.08 (−1.58)	1.06 (0.85)
Immune system disorders	27	1.03 (0.71–1.51)	1.03 (0.03)	0.04 (−1.62)	1.03 (0.71)
Respiratory, thoracic and mediastinal disorders	74	0.76 (0.60–0.96)	0.77 (5.49)	−0.38 (−2.05)	0.77 (0.61)
Hepatobiliary disorders	17	0.97 (0.60–1.56)	0.97 (0.019)	−0.05 (−1.71)	0.97 (0.60)
Product issues	30	0.80 (0.56–1.14)	0.80 (1.51)	−0.32 (−1.99)	0.80 (0.56)
Musculoskeletal and connective tissue disorders	74	0.67 (0.53–0.84)	0.68 (11.62)	−0.55 (−2.22)	0.68 (0.54)
Renal and urinary disorders	33	0.74 (0.53–1.05)	0.75 (2.88)	−0.42 (−2.09)	0.75 (0.53)
Gastrointestinal disorders	111	0.63 (0.52–0.76)	0.65 (22.64)	−0.62 (−2.29)	0.65 (0.54)
Nervous system disorders	103	0.63 (0.52–0.77)	0.65 (21.42)	−0.63 (−2.29)	0.65 (0.53)
Reproductive system and breast disorders	12	0.85 (0.48–1.50)	0.85 (0.31)	−0.23 (−1.90)	0.85 (0.48)
Investigations	64	0.50 (0.39–0.64)	0.51 (31.05)	−0.96 (−2.62)	0.51 (0.40)
Metabolism and nutrition disorders	20	0.47 (0.31-0.74)	0.48 (11.50)	−1.06 (−2.73)	0.48 (0.31)
Blood and lymphatic system disorders	17	0.47 (0.29–0.76)	0.48 (9.99)	−1.07 (−2.74)	0.48 (0.30)
Social circumstances	6	0.60 (0.27–1.33)	0.60 (1.61)	−0.74 (−2.40)	0.60 (0.27)
Endocrine disorders	4	0.71 (0.27–1.90)	0.71 (0.46)	−0.49 (−2.15)	0.71 (0.27)
Congenital, familial and genetic disorders	4	0.67 (0.25–1.77)	0.67 (0.67)	−0.59 (−2.25)	0.67 (0.25)
Vascular disorders	15	0.37 (0.22−0.62)	0.37 (15.97)	−1.42 (−3.08)	0.37 (0.23)
Psychiatric disorders	24	0.20 (0.13–0.30)	0.21 (75.34)	−2.25 (−3.92)	0.21 (0.14)
Eye disorders	9	0.22 (0.11–0.42)	0.22 (24.84)	−2.17 (−3.83)	0.22 (0.12)
Ear and labyrinth disorders	3	0.34 (0.11–1.04)	0.34 (3.92)	−1.57 (−3.24)	0.34 (0.11)
Pregnancy, puerperium and perinatal condition	1	0.13 (0.02–0.89)	0.13 (6.12)	−2.99 (−4.66)	0.13 (0.02)

^a^ SOCs are positive.

^b^ The SOC satisfies in four algorithm simultaneously.

^c^ The values are positive signals in algorithm.

ADRs, adverse drug reactions; TIL, Tildrakizumab; SOC, system organ class; ROR, reporting odds ratio; CI, confidence interval; PRR, proportional reporting ratio; χ^2^, chi-squared; IC, information component; IC025, the lower limit of 95% CI of the IC; EBGM, empirical Bayesian geometric mean, EBGM05, the lower limit of 95% CI of EBGM. *N: total cases of Tildrakizumab reporting.

**TABLE 3:** Signal strength of ADRs of TIL at the PTs level in FAERS database.

Preferred terms (PTs)	N*	ROR (95% two-sided CI)	PRR (χ^2^)	IC (IC025)	EBGM (EB 05)
SOC: Surgical and medical procedures					
Therapy cessation^a^	13	9.51 (6.31–14.35)	9.42 (173.19)	3.23 (1.57)	9.41 (6.67)
Therapy interrupted^a^	13	4.30 (2.49–7.42)	4.28 (32.73)	2.10 (0.43)	4.28 (2.71)
SOC: Infections and infestations					
Pneumonia^b^	30	2.71 (1.89–3.89)	2.69 (31.97)	1.43 (−0.24)	2.69 (1.99)
Urinary Tract Infection^a^ ^,^ ^b^	29	4.92 (3.41–7.10)	4.87 (89.34)	2.28 (0.62)	4.87 (3.58)
Bronchitis^a^ ^,^ ^b^	12	5.16 (2.92–9.10)	5.13 (39.98)	2.36 (0.69)	5.13 (3.19)
Cellulitis^a^ ^,^ ^b^	11	6.58 (3.64–11.90)	6.55 (51.75)	2.71 (1.04)	6.55 (3.99)
Sinusitis^b^	10	2.84 (1.53–5.29)	2.83 (11.86)	1.50 (−0.17)	2.83 (1.68)
Herpes Zoster^a^ ^,^ ^b^	9	4.36 (2.27–8.39)	4.35 (23.20)	2.11 (0.45)	4.34 (2.51)
Upper Respiratory Tract Infection^a^ ^,^ ^b^	9	5.95 (3.09–11.45)	5.93 (36.87)	2.57 (0.90)	5.92 (3.42)
Vulvovaginal Candidiasis^a^ ^,^ ^b^	5	121.70 (50.39–293.92)	121.42 (591.50)	6.91 (5.24)	120.28 (57.52)
Diverticulitis^b^	4	4.04 (1.51–10.76)	4.03 (9.11)	2.01 (0.34)	4.03 (1.77)
Respiratory Tract Infection^a^ ^,^ ^b^	4	4.37 (1.64–11.66)	4.36 (10.38)	2.13 (0.46)	4.36 (1.92)
Tuberculosis^a^ ^,^ ^b^	4	9.24 (3.46–24.66)	9.23 (29.33)	3.21 (1.54)	9.22 (4.06)
Coronavirus Infection^a^	4	8.40 (3.15–22.40)	8.38 (26.00)	3.07 (1.40)	8.38 (3.69)
Ear Infection	3	3.12 (1.01–9.71)	3.12 (4.33)	1.64 (−0.02)	3.12 (1.21)
Herpes Simplex^a^	3	18.48 (5.95–57.38)	18.45 (49.45)	4.20 (2.54)	18.43 (7.14)
SOC: Injury, poisoning and procedural complications					
Exposure During Pregnancy^a^ ^,^ ^b^	12	5.03 (2.85–8.87)	5.01 (38.50)	2.32 (0.66)	5.00 (3.11)
Meniscus Injury^b^	3	12.58 (4.05–39.05)	12.56 (31.90)	3.65 (1.98)	12.55 (4.86)
SOC: Skin and subcutaneous tissue disorders					
Psoriasis^a^ ^,^ ^b^	57	11.44 (8.79–14.88)	11.16 (528.01)	3.48 (1.81)	11.15 (8.95)
Pruritus^a^ ^,^ ^b^	31	2.41 (1.69–3.44)	2.39 (25.19)	1.26 (−0.41)	2.39 (1.78)
Rash^a^ ^,^ ^b^	28	1.81 (1.25–2.63)	1.80 (10.11)	0.85 (−0.82)	1.80 (1.32)
SOC: Cardiac disorders					
Atrial Fibrillation^a^	11	3.43 (1.90–6.21)	3.42 (18.85)	1.77 (0.11)	3.42 (2.08)
Myocardial Infarction^a^ ^,^ ^b^	10	3.16 (1.70–5.87)	3.15 (14.66)	1.65 (−0.01)	3.15 (1.87)
Aortic Valve Incompetence^a^	3	31.99 (10.29–99.41)	31.94 (89.71)	4.99 (3.32)	31.87 (12.34)
SOC: General disorders and administration site conditions					
Drug Ineffective	138	2.90 (2.44–3.45)	2.78 (160.52)	1.47 (−0.19)	2.78 (2.40)
Condition Aggravated	30	2.51 (1.75–3.60)	2.49 (26.89)	1.32 (−0.35)	2.49 (1.84)
Therapeutic Response Decreased^a^	13	7.24 (4.19–12.48)	7.20 (69.39)	2.85 (1.18)	7.19 (4.56)
Disease Recurrence^a^	8	3.65 (1.82–7.31)	3.64 (15.32)	1.86 (0.20)	3.64 (2.04)
Therapy Non-Responder	7	3.45 (1.64–7.25)	3.44 (12.14)	1.78 (0.12)	3.44 (1.85)
Therapy Partial Responder^a^	5	8.12 (3.38–19.54)	8.10 (31.13)	3.02 (1.35)	8.10 (3.89)
Injection Site Urticaria^a^ ^,^ ^b^	4	5.88 (2.20–15.68)	5.87 (16.16)	2.55 (0.89)	5.87 (2.58)
Therapeutic Response Unexpected	4	2.77 (1.04–7.40)	2.77 (4.53)	1.47 (−0.20)	2.77 (1.22)

^a^ PTs meet the four algorithms.

^b^ ADRs on the basis of Tildrakizumab mechanism of action or anticipated from pre-marketing pivotal trials.

ADRs, adverse drug reactions; TIL, Tildrakizumab; SOC, system organ class; PTs, preferred terms; ROR, reporting odds ratio; CI, confidence interval; PRR, proportional reporting ratio; χ^2^, chi-squared; IC, information component, IC025, the lower limit of 95% CI of the IC; EBGM, empirical Bayesian geometric mean; EBGM 05, the lower limit of 95% CI of EBGM; N^*^, total cases of Tildrakizumab reporting.

### 3.3 Time-to-event onset analysis

**FIGURE 5:**
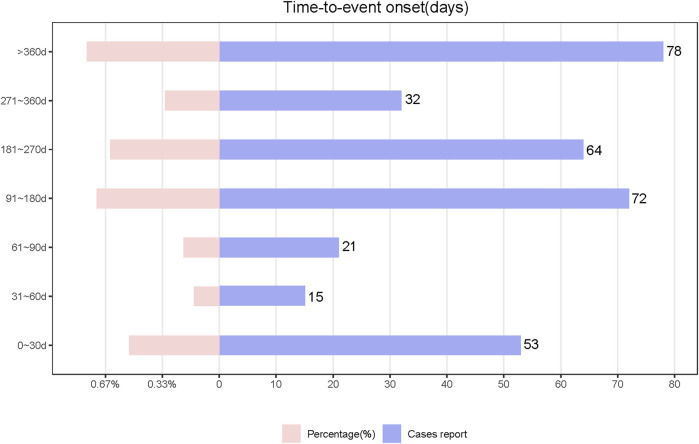
Time of occurrence of Tildrakizumab-associated ADRs.

**FIGURE 6:**
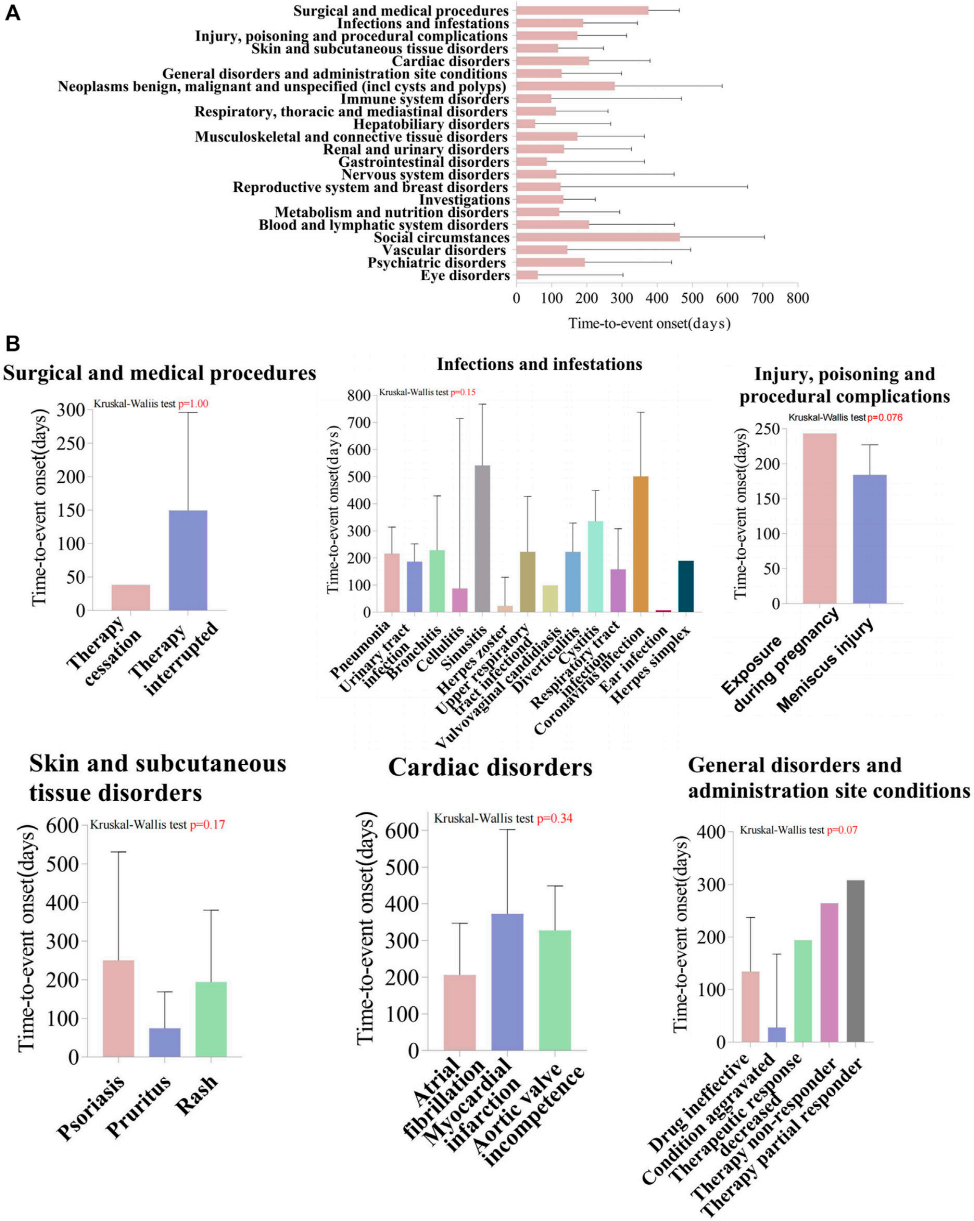
Time-to-event onset (TTO) analysis of ADRs at the SOC and PT levels. **(A)** Box plot of the TTO at the SOC level for Tildrakizumab. The blue box represents the median, while the black bar indicates the interquartile range. **(B)** Comparing the TTO among PTs within the SOC level, using Kruskal–Wallis test to detect whether the medians within each group are significantly different from each other. The box represents the median, while the black bar indicates the interquartile range. SOC, System Organ Class; PTs, preferred terms.

We analyzed TIL-induced 335 ADRs using the TTO reported after excluding missing data and reporting errors (Figure 5). The median time to onset was 194 days, with the IQR range from 84 to 329 days. The number of ADR reports was higher in the first month after administration (n = 53, percentage = 15.82%). The growth rate of the ADRs reports in the second and third months was −71.7% and 40%, respectively. The number of ADRs decreased each quarter over time with the following numbers and growth rates: N = 89; N = 72, growth rate = −19.1%; N = 64, growth rate = −11.1%; N = 32, growth rate = −50%. After 1 year of TIL treatment, the incidence of ADRs was 23.28% (n = 78). Analyzed by the Kolmogorov-Smirnov Goodness of Fit test (*p* = 0.93) and Weibull test (Supplementary Table S4), β = 0.89 (95% CI:CI:0.80–0.97),andη = 269.56 (95% CI:231.36–307.76), indicating that the trend of ADRs was towards the early type. This implies that the risk of ADRs increases after medication and then decreases over time, which is also consistent with the trend in growth rates. Whereas about 63.2% of ADRs occurred within 269.56 days, which suggests that monitoring of TIL ADRs should be long term. Top 5 SOCs with earliest onset to ADRs after medication were observed for hepatobiliary disorders (median = 53), eye disorders (median = 60.5), gastrointestinal disorders (median = 86), immune system disorders (median = 99), and respiratory, thoracic, and mediastinal disorders (median = 112), as shown in Figure 6A, which all occurred 2–4 months after dosing. We analyzed PTs within six groups of SOCs with positive signals, and the Kruskal–Wallis test determined that there was no significant differences in the timing within the groups (Figure 6B).

## 4 Disscusion

TIL is approved for the treatment of moderate to severe plaque psoriasis in the United States, European Union, Australia, and Japan (Lebwohl et al., 2021). As an IL-23 antagonist, it was approved for chronic plaque psoriasis in 2018. We observed a significant increase in ADRs from an initial 13 to 392 cases reported in 2023 over 5 years. We consider that the notable rise in ADRs during this period to the authorisation of TIL, which led to an increase in the number of prescriptions and an expanded of the user base. Based on the FAERS spontaneous reporting system, we collected a total of 1,177 post-marketing-induced ADRs for TIL, the highest number of TIL ADR reports collected to date. ADRs can lead to discontinuation of medication or non-adherece by the patient, which can compromise effective disease control. Therefore, a thorough understanding of the side effects of TIL is essential for patient management (Kolli et al., 2019). The age of onset of psoriasis is centred on 16-22 and 57–60 years, and is common in Caucasians and high-income countries. This is consistent with the reported age distribution (18–64 years) and the reporting areas mainly in Caucasian developed countries (Di Lernia and Goldust, 2018; Griffiths et al., 2021). Previous studies have shown that TIL is more effective in patients with lower body weight (Papp et al., 2019). Based on the data collected, patients weighing less than 50 kg also had the lowest rate of ADRs. In our findings, although there was no significant gender difference in the incidence of psoriasis, the proportion of ADRs was slightly higher in male patients than in females (Griffiths et al., 2021).

Although health professionals comprised 25.4% of the reporting population, the proportion was higher among non-professional consumers (52.2%). Considering the phenomenon that “surgical and medical procedures” have the highest SOC signal intensity, the increase in detection of ADRs may be due to the simplicity of these procedures, such as therapy cessation and therapy interruption, leading to more frequent reporting by patients without relevant medical expertise. This indicates the need for enhanced educational interventions for FAERS reporters to improve reporting accuracy and reporting rate (Cervantes-Arellano et al., 2024). Meanwhile, We know that treatment is often stopped or interrupted in the real world for a variety of reasons. Interruption of TIL treatment was also modelled in the previous reSURFACE one trial, which showed a decrease in disease control after interruption. However, approximately 85% of patients who relapsed and resumed TIL achieved PASI 75, which provides an important guide to restoring confidence in treatment for patients who interrupt therapy (Kimball et al., 2020).

Due to their high targeting of inflammation, biologics paradoxically lead to an enhanced immune response, which is particularly evident with anti-IL-23p19 and anti-IL-17 drugs (Griffiths et al., 2021). In addition, the use of TIL reduces the production of psoriasis-associated lymphocyte cytokines (e.g., IL-17), which normally induce the production of antimicrobial peptides (e.g.,β-defensin and S100A7) against fungi, thereby reducing fungal resistance (Ghoreschi et al., 2021). In addition to infections mentioned in relevant trials and manufacturer’s documents, such as upper respiratory tract infection, respiratory tract infection, tuberculosis, cellulitis, diverticulitis, pneumonia, urinary tract infection, bronchitis, herpes zoster, vulvovaginal candidiasis (Thaci et al., 2021; Egeberg et al., 2023), coronavirus infection (ROR = 8.40) and herpes simplex (ROR = 18.48) also exhibit significant signals, which have not been emphasized in previous RCT studies or retrospective cases. Clinicians who are treating plaque-type psoriasis with TIL in patients with plaque psoriasis should monitor the incidence of these infections and be aware of gender differences in incidence. IL-23 antagonists act on similar inflammatory pathways as IL-17 antagonists. Given that IL-17 antagonists exacerbate inflammatory bowel disease (IBD) in the real world, relevant studies have begun to examine whether the use of TIL is associated with the development or exacerbation of IBD. However, the phase 2b (P05495, NCT01225731) and two phase 3 (reSURFACE 1; reSURFACE 2) trial studies found that TIL did not induce or exacerbate IBD in patients with psoriasis (Gooderham et al., 2019). Moreover, in a pooled analysis after 148 weeks of reSURFACE one and reSURFACE, the incidence of Crohn’s disease (CD) was 0.05 events per 100 person-years (Reich et al., 2020), further confirming the accuracy of previous studies. Supplementary Table S5 showed diarrhoea (N = 29, ROR: 1.29, 95% CI: 0.89–1.86), IBD (N = 2, ROR: 11.26, 95% CI: 2.81–45.08), and CD (N = 1, ROR: 0.42, 95% CI: 0.06–2.98), none of which showed a positive signal. Furthermore, IL-23 antagonists have shown good potential in the treatment of IBD, as evidenced by the fact that Ustekinumab has been approved by the FDA for the treatment of CD. However, the relationship between TIL and IBD needs to be confirmed by further studies.

We observed that common adverse events such as nasopharyngitis, diarrhoea and headache, which have been documented in previous trials and various retrospective case analyses, did not show a positive signal in disproportionality analysis (Egeberg et al., 2023). This suggests that these ADRs are quite common in the FAERS database, preventing the manifestation of disproportionality, and therefore remain unnoticed in disproportionality analyses. This is known as the masking effect or cloaking effect, and also affects BCPNN and EBGM(30). Accurate assessment of TIL-associated ADRs requires a combination of extensive experimental data and real-world analyses.

Significant signals are observed with “Exposure during pregnancy”. Subcutaneous administration of 100 mg/kg to pregnant monkeys did not produce treatment-related effects on the developing fetuses (Santostefano et al., 2019). Post hoc analyses of nine clinical trials of TIL did not identify any birth defects or abnormalities in the infants. The rate of miscarriage (14%) was similar to that of the general population (12%–15%) (Haycraft et al., 2020). IgG1 can cross the placental barrier efficiently by Fc receptor (FcRn)-mediated transport from 13th week of pregnancy (Palmeira et al., 2012). The known half-life of TIL is 23 days (Pharma, 2022), suggesting that it has the potential to cross the placental barrier. Given the significant signals calculated to result from exposure to TIL during pregnancy and the limited data available on the use of TIL in pregnant women, we agree with Haycraft K et al. that caution should be exercised in the use of TIL in women of childbearing potential (Haycraft et al., 2020). Skin adverse reactions such as psoriasis, pruritus, rash, and injection site urticaria have also been observed in previous studies, which is consistent with our findings. It is well known that patients with psoriasis are more prone to cardiovascular disease (Ozden et al., 2016), so cardiac disorder is also a noteworthy SOC. In this study, they also showed strong signals including atrial fibrillation, myocardial infarction, and aortic valve incompetence. During the 5-year treatment follow-up, the common cardiac disorder was acute myocardial infarction, which led to one fatal case (Macaluso et al., 2019). However, based on our findings, atrial fibrillation and aortic valve incompetence are also noteworthy. No significant positive signals were observed for neoplasms, benign tumours, malignant tumours and tumours of unknown origin (including cysts and polyps), which is consistent with the previously low incidence of neoplasms.

Although we performed a detailed analysis of TIL-associated ADRs in FAERS, the study still has some omissions due to the characteristics of the FAERS data itself. Firstly, the absence of prescribing information for TIL, including information on drug dosage and administration, may have led to discrepancies in ADRs; secondly, some ADRs may have been reported by non-healthcare professionals, leading to possible inaccuracies in the characterisation of ADRs; thirdly, the FAERS database comprises mainly data from Western countries, with less self-reported data from Third World countries. Fourthly, data sources that use joint analysis of multiple databases, such as the British Association of Dermatologists Biologics and Immunomodulators and Germany’s PsoBest, were not included. Finally, each method for calculating ADRs has its own advantages and disadvantages. Although ROR is the most common and straightforward FAERS-based analysis (Rana et al., 2021), its performance, along with that of PRR, declines in the presence of the innocent bystander effect (i.e., concomitant use of a specific drug with the drug that caused the ADR, leading to incorrect attribution of the ADR to the specific drug) (Dijkstra et al., 2020). On the other hand, BCPNN and MGPS tend to be overly conservative and may ignore ADRs (Noguchi et al., 2021). Nonetheless, FAERS remains the largest ADR database in the world. It is crucial to understand that signal detection should not be interpreted as a direct causal relationship, but rather the association between drugs and ADRs should be analysed to provide real-world evidence. Therefore, it is important to use this database to assess the relationship between TIL and its ADRs to provide a more comprehensive perspective on TIL vigilance analyses.

## 5 Conclusion

Our study used the data mining algorithms ROR, PRR, IC and EBGM to detect ADR signals in long-term treatment with TIL, and identified a number of unreported ADRs associated with TIL, which could be beneficial for future risk detection and clinical use of the drug.

## Data Availability

The original contributions presented in the study are included in the article/Supplementary Material, further inquiries can be directed to the corresponding author.

## Abbreviations

TIL, Tildrakizumab; IL-23, Interleukin-23; IL-17, Interleukin-17; ADRs, Adverse Drug Reactions; PTs, Preferred Terms; SOC, System Organ Class; FDA, Food and Drug Administration; FAERS, FDA Adverse Event Reporting Database; TTO, Time-to-event onset; ROR, Reporting Odds Ratios; IC, Confidence Interval; IBD, Inflammatory Bowel Disease; CD, Crohn’s disease.
