# Supplementary material for: Safety of tildrakizumab: a disproportionality analysis based on the FDA adverse event reporting system (FAERS) database from 2018–2023
Source: Front Pharmacol. 2024 Jul 10;15:1420478. doi: 10.3389/fphar.2024.1420478 (PMC11267582; doi:10.3389/fphar.2024.1420478)
Supplement: Supplementary file 1 [file Table1.DOCX]

Table S1 Two-by-two contingency table for disproportionality analyses

| Drugs | Target adverse reaction | Other adverse reactions | Total |
| --- | --- | --- | --- |
| Selected drug | a | b | a+b |
| All other drugs | c | d | c+d |
| Total | a+c | b+d | n=a+c+b+d |
