# Supplementary material for: Safety of tildrakizumab: a disproportionality analysis based on the FDA adverse event reporting system (FAERS) database from 2018–2023
Source: Front Pharmacol. 2024 Jul 10;15:1420478. doi: 10.3389/fphar.2024.1420478 (PMC11267582; doi:10.3389/fphar.2024.1420478)
Supplement: Supplementary file 2 [file Table2.DOCX]

Table S2. Four algorithms used for signal detection.

| Algorithms^✝^ | | Equation^#^ | Criteria^Ψ^ |
| --- | --- | --- | --- |
| ROR | ROR=ad/b/c | | lower limit of 95% CI>1, N≥3 |
|  | 95%CI=e^ln(ROR)±1.96(1/a+1/b+1/c+1/d)^0.5^ | |  |
| PRR | PRR=a(c+d)/c/(a+b) | | PRR≥2, χ^2^≥4, N≥3 |
|  | χ^2^=[(ad-bc)^2](a+b+c+d)/[(a+b)(c+d)(a+c)(b+d)] | |  |
| BCPNN | IC=log_2_^a(a+b+c+d) (a+c) (a+b)^ | | IC025>0 |
|  | 95%CI= E(IC) ± 2V(IC)^0.5 | |  |
| MGPS | EBGM=a(a+b+c+d)/(a+c)/(a+b) | | EBGM05>2 |
|  | 95%CI=eln^(EBGM)±1.96(1/a+1/b+1/c+1/d)^0.5^ | |  |

✝: ROR, reporting odds ratio; PRR: proportional reporting ratio; BCPNN: Bayesian confidence prop-agation neural network; MGPS: multi-item gamma passion shrinker; **#**: a, number of reports containing both the target drug and target adverse drug reaction; b, number of reports containing other adverse drug reaction of the target drug; c, number of reports containing the target adverse drug reaction of other drugs; d, number of reports containing other drugs and other adverse drug reactions; 95% CI: 95% confidence interval; χ^2:^ chi-squared; IC: information component, E(IC): the IC expectations, V(IC): the variance of IC; EBGM:empirical Bayesian geometric mean; Ψ: IC025: the lower limit of 95% CI of the IC, EBGM05:the lower limit of 95% CI of EBGM, N: the number of cases reporting.
