# Supplementary material for: Safety of tildrakizumab: a disproportionality analysis based on the FDA adverse event reporting system (FAERS) database from 2018–2023
Source: Front Pharmacol. 2024 Jul 10;15:1420478. doi: 10.3389/fphar.2024.1420478 (PMC11267582; doi:10.3389/fphar.2024.1420478)
Supplement: Supplementary file 3 [file Table3.DOCX]

Supplementary Table 3 The PTs of the male and female subgroups

| PT | A1 | A2 | B1 | B2 | ROR(95%CI) | P_value |
| --- | --- | --- | --- | --- | --- | --- |
| Drug Ineffective | 57 | 878 | 45 | 913 | 0.76(0.51-1.13) | 0.21 |
| Psoriasis | 24 | 911 | 29 | 929 | 0.73(0.34-1.55) | 0.64 |
| Condition Aggravated | 16 | 919 | 12 | 946 | 2.62(0.69-9.89) | 0.52 |
| Pruritus | 15 | 920 | 13 | 945 | 0.98(0.24-3.91) | 0.80 |
| Urinary Tract Infection | 13 | 922 | 16 | 942 | 0.73(0.16-3.28) | 0.76 |
| Therapy Cessation | 12 | 923 | 10 | 948 | 1.20(0.58-2.52) | 0.79 |
| Celluitis | 6 | 929 | 5 | 953 | 1.96(0.59-6.53) | 0.97 |
| Upper Respiratory Tract Infection | 5 | 930 | 4 | 954 | 0.81(0.25-2.67) | 0.97 |
| Therapy Non-Responder | 4 | 931 | 3 | 955 | 0.78(0.21-2.91) | 0.97 |
| Bronchitis | 4 | 931 | 8 | 950 | 1.18(0.68-2.05) | 0.41 |
| Disease Recurrence | 4 | 931 | 4 | 954 | 0.84(0.40-1.78) | 1.00 |
| Therapeutic Response Decrease | 3 | 932 | 8 | 950 | 0.81(0.35-1.89) | 0.24 |

A1, target ADRs of male using Tildrakizumab; A2, other ADRs of male using Tildrakizumab; B1, target ADRs of female using Tildrakizumab; B2, other ADRs of female using Tildrakizumab.
