# Supplementary material for: Safety of tildrakizumab: a disproportionality analysis based on the FDA adverse event reporting system (FAERS) database from 2018–2023
Source: Front Pharmacol. 2024 Jul 10;15:1420478. doi: 10.3389/fphar.2024.1420478 (PMC11267582; doi:10.3389/fphar.2024.1420478)
Supplement: Supplementary file 4 [file Table4.DOCX]

Supplementary Table 4 Time-to-event onset of Tildrakizumab.

| Drug | N^*^ | Median(d)(25%-75%) | p^†^ | Scale parameter:η(95%CI) | Shape parameter:β(95%CI) | Type^＃^ |
| --- | --- | --- | --- | --- | --- | --- |
| Tildrakizumab | 335 | 194(84~329) | 0.93 | 269.56(231.36-307.76) | 0.89(0.80-0.97) | Early failure |

N*: cases report of Tildrakizumab-related ADRs; p^✝^: calculated by Kolmogorov-Smirnov Goodness of Fit test, p > 0.05 indicates that the data conforms to the Weibull test. Type^#^: 95% CI of β included 1, the hazard is constant over time (random failure type), the lower limit of the 95% CI of β is >1, the hazard increase over time (wear-failure type), the upper limit of the 95% CI of β is <1, the hazard decreased over time (early failure type); CI: Confidence Interval.
